# Application of a simulation model to the prognosis of material loss in wood processing

**DOI:** 10.1371/journal.pone.0246325

**Published:** 2021-02-02

**Authors:** Krzysztof Żywicki, Tomasz Bartkowiak, Agnieszka Kujawińska

**Affiliations:** Faculty of Mechanical Engineering, Poznan University of Technology, Poznan, Poland; Ton Duc Thang University, VIET NAM

## Abstract

The article discusses the influence of a sample size on the credibility of a simulation model created for the estimation of material loss in the production of a middle layer of a wooden floorboard. The study was conducted in a production company operating in the wood processing industry. Geometric characteristics of input material were captured and used to derive statistical distributions, which were then included in the simulation model. The conducted experiments indicated that the quality of the simulation model was significantly affected by the quality and quantity of the sample, on the basis of which the stochastic model is estimated. It was shown that small sample for wood processing data was insufficient to capture process variability. On the other hand, excessive sample size (80 or more observations) for the material with high natural geometric variability, involves taking into account outliers, which may lower the overall prognostic quality of the simulation model. Based on the conducted simulation experiments, the recommended sample size which allows development of a reliable model for estimation of material loss in the analyzed manufacturing process, ranges from 40 to 60 measurements.

## Introduction

A common characteristic of all entities manufacturing wood products is the structure of their production cost, of which raw material forms a significant share. In the sawmill industry, raw material accounts for about 70% total cost [1]. Similar figures are common in production of the components of laminated floors, e.g. for sawing wood on frame, band or circular saws [2, 3].

Most of the popular timber trees, such as pine or oak, increase their prices by 8% annually [4]. In striving for a competitive position, businesses operating in the timber sector try to not pass on their expenses to consumers. Hence, the minimization of raw material cost by optimization of production and the development of new technologies has become a main challenge in the wood industry. Mechanical processing is the fundamental technology for timber and is not free of material loss [5]. Material loss is usually caused by over-estimated machining allowances. The problem of excessive material loss exists in the manufacturing of laminated floorboard. According to Orlowski, in the production of a top layer, which is made of oak, the raw material cost exceeds 80% of total cost of manufacturing, while the tool cost for a frame and band saw constitute 6% and 5% respectively [2]. In the production of slats, it is important to locate and analyze the source of loss and to provide the most effective solution for minimizing them.

In the processing of wood raw material, it is important to determine demand for the input material of a specific quality necessary to produce a certain number of items. Timber is characterized by the high heterogeneity of its structure and the lack of dimensional stability. For this reason, it is important to search for solutions in the fields of demand planning and production control. For this purpose, simulation methods that allow analysis and evaluation of various principles, methods or processes are frequently used which [6–8]. The term "simulation" is associated with the imitation of a real situation, real objects and their interconnections [9, 10]. Simulation is a research method and enables analysis and evaluation of potential solutions and changes outside real processes [11]. In a simplified way, the simulation is usually conducted in three steps [12]:

- designing a simulation model of a real process or system,
- conducting experiments using a simulation model,
- using the obtained results to improve the actual system or process.

Simulation models of manufacturing processes are created to reduce the risk of failure when making significant changes to the actual process or when designing a new process to develop the best variant of production organizing. Simulations also test new rules, allow insight into complex structure, analyze production indicators, and collect information and knowledge without interfering with the actual process [13–15]. Most the simulative studies, which concern material requirement planning, focus on the organizational aspects of production, e.g. schedule instability [16, 17], service level [17] or cost [18–20]. Such studies concentrate on disturbances in frequencies of material supply or quality of input material understood as binary (good or bad). In the wood industry, where geometric instability of material is an inherent part of the manufacturing, there have been endeavours to investigate its overall effect on the production. Neda Sowlati summarized modelling methods of assessing the productivity and efficiency of the Canadian wood industry [21]. Mayer et al. presented a similar study of German companies [22]. These macro models and simulations do not analyze, in detail, the effects of dimensional variability on a particular manufacturing process. However, this, is considered as an important problem to the production planner and controller, who handles this effect in the scheduling and calculation of cost or price of final product. There are two main problems identified here in this study. Because of the randomness of material geometric characteristics, proper sampling becomes a key factor to accurately estimate the effects on productivity. Sampling more data requires additional resources for its collection and analysis and does not necessarily induce higher credibility of the models based on that data. On the other hand, collecting a small amount of information might lead to significant under- or overestimation errors. Another issue is how to translate geometric dimensions of the input material into an exact amount of output product. These two problems are addressed in this study.

Most common engineering and scheduling problems involve handling small samples or poor quality of collected information. Therefore, it is important to effectively generate, analyze and extract useful information from what is available from uncertain systems with partially known data. So, system operational behaviour and its laws of evolution can be correctly described, monitored and forecasted within acceptable uncertainty. This problem is addressed here through a simulative study, which facilitate determination of proper sampling size for the given manufacturing problem.

This article presents the results of research aimed at determining the effectiveness of the application of simulation methods to forecast the material loss in the processing of wood material. The simulation model of the analyzed production process was developed and the results were validated with real data.

## Method and materials

### Description of the technological process

**Fig 1:**
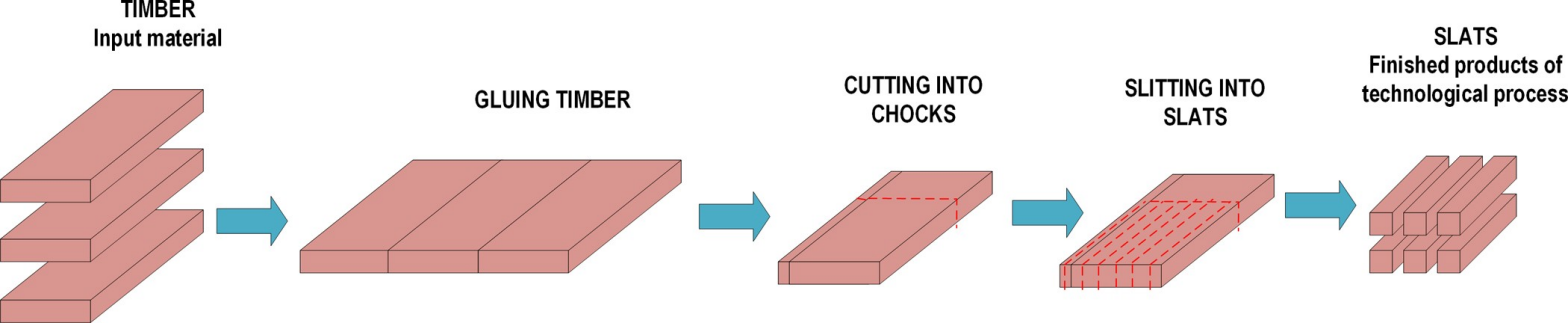
Scheme of slats manufacturing process.

This research is focused on the production process of slats constituting the main structural element of the middle layer of a wooden floor board. The input material is softwood logs. The process consists of the following operations: gluing cut timbers, cutting the glued "tape" of cut timbers into the so-called chocks, which are then cut into slats (Fig 1).

Material loss arises in the course of subsequent technological operations. Measuring the ratio between input (timbers) and output (slats) volume allows estimation of loss in the range of 30–40%. It should be emphasized, however, that the results of this analysis are based on the theoretical data, i.e. material consumption standards, and do not take into account the actual dimensions of the material. Such large differences make it difficult to calculate the amount of input material needed to produce a specific quantity of finished product. The main reason for such a low accuracy in determining the demand for the input material is the instability of the geometric dimensions of the input material and its inhomogeneous internal structure. To find a solution that would increase the accuracy of calculating material loss in the process, research was conducted by means of simulation methods.

### Purpose of the study

**Fig 2:**
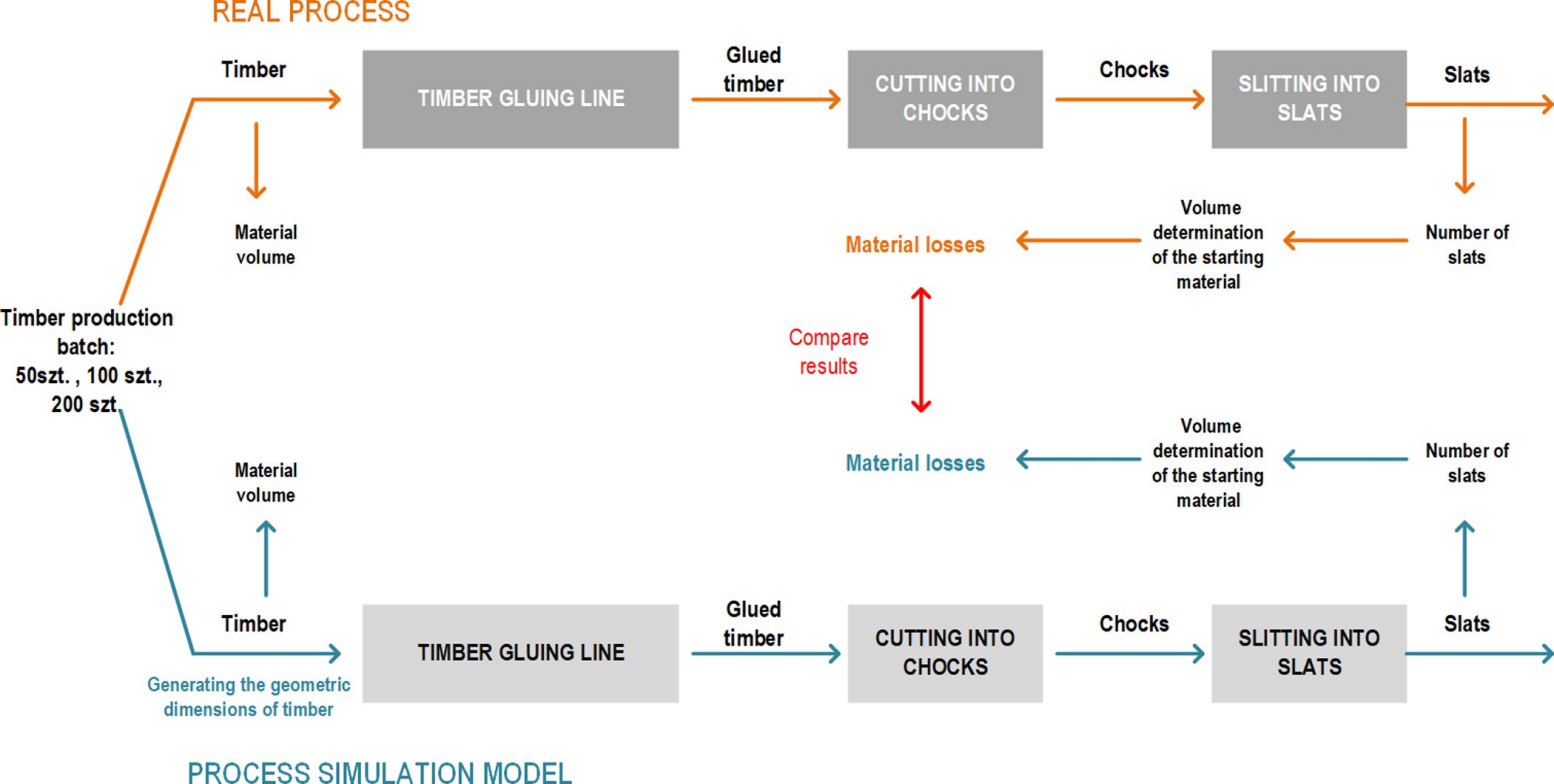
The research and experiments plan to determine the effectiveness of forecasting material loss.

The aim of the research was to determine the possibility of using simulation modelling to forecast and calculate material loss in the discussed technological process. The tests were conducted in discrete event simulation environment. Validation of the simulation model was performed by comparing the results of simulation experiments with the actual results obtained in the production process. The actual measurements were performed manually using validated measuring instruments. The tests were carried out for two types of input material of different geometric dimensions: 1300 mm and 1800 mm long timbers (Fig 2).

The research plan involved:

- development of a simulation model
- preparation of input data for the simulation model,
- conducting simulation experiments,
- measurements and calculation of real data in the technological process,
- comparison of the results obtained from the simulation model with real data.

The developed simulation model covered all stages and characteristics of the actual production process. It was based on 6 variants of input data, which included sets of geometric dimensions of timbers and intermediate semi-finished elements (length, width and thickness). These variants differed in the size of the sample constituting the basis for generating the datasets constituting the input for the simulation experiments and amounted to: 10, 20, 40, 60, 80, and 100 items.

Based on this sample data, the volume of the material was estimated, by applying statistical fitting of known distributions, and then used to calculate material loss and the final number of produced slats. Geometric characteristics were obtained as a result of measurements in the actual technological process.

The conducted simulation experiments were aimed at determining material loss for three production batches with input material sample sizes of 50, 100 and 200 pieces. Forecasting material loss in the simulation model was performed in 30 repetitions for each variant of the input dataset.

Validation of the simulation model was done by comparing the simulation results with the actual data. The actual data was obtained as a result of manual measurements of the geometric properties of the material. Measurements were carried out for three batches of 50, 100, and 200 pieces, in similarity to the simulation experiments. The obtained data allowed calculation of the volume of the material and determination of the resulting loss. These calculations were compared with the results obtained during the simulation experiments.

### Calculation of material loss

**Fig 3:**
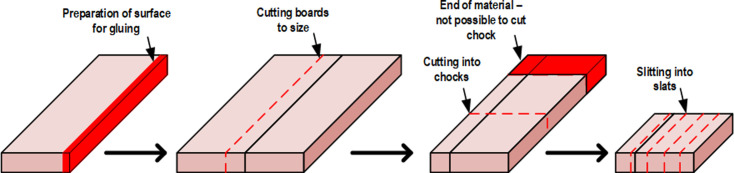
Factors contributing to material loss in the discussed process.

The technological process generates loss in the volume of the material related primarily to the geometric heterogeneity of the input material (timbers) and technological loss resulting from the use of processing tools. Fig 3 summarizes contributing factors.

Total material loss can be expressed as a sum of loss of particular technological operations in the following form:

$$L_{t}=L_{b}+L_{c}+L_{l}$$

where:

*L*_*t*_−total material loss of input material [m^3^],

*L*_*b*_−material loss of preparing surface of timbers for gluing and cutting into the boards[m^3^],

*L*_*c*_−material loss of cutting the tape into chocks [m^3^],

*L*_*l*_−material loss of cutting the chocks into slats [m^3^].

The material loss created after gluing timbers was calculated by the following formula:

$$L_{b}=L_{t}-\left[\left(s_{t}-s_{tk}\right)\cdot g_{t}\cdot d_{t}\right]$$

where:

*V*_*t*_−volume of timbers (input material) [m^3^],

*s*_*t*_−width of timbers [m],

*s*_*tk*_−width of tape [m],

*g*_*t*_−thickness of timbers [m],

*d*_*t*_−length of timbers [m].

The operation of cutting the tape into the chocks causes material loss that can be determined by the relationship:

$$L_{c}=V_{tk}-\sum_{i=1}^{n}V_{w_{i}}$$

where:

*V*_*tk*_−tape volume [m^3^],

*V*_*w*_−single chock volume [m^3^],

n–number of chocks cut from the tape.

Volume of a cut chock is expressed by the formula:

$$V_{w}=s_{w}\cdot g_{w}\cdot d_{w}$$

*s*_*w*_−chock width [m],

*g*_*w*_−chock thickness [m],

*d*_*w*_−chock length [m].

In the final operation, slats are cut from chocks, which adds the following loss:

$$L_{l}=V_{w}-\sum_{i=1}^{n}V_{l_{i}}$$

where:

*V*_*w*_−chock volume [m^3^],

*V*_*l*_−slats volume [m^3^],

n–number of slats cut from chocks.

Slat volume can be noted as:

$$V_{l}=s_{l}\cdot g_{l}\cdot d_{l}$$

where:

*V*_*l*_−slat volume [m^3^],

*s*_*l*_−slat width [m],

*g*_*l*_−slat thickness [m],

*d*_*l*_*−*slat length [m].

Taking into account the volume of the input material and the number of chocks and slats obtained at a given operation, it is possible to calculate the loss generated in the production process.

## Simulation model

### Description of simulation model

**Fig 4:**
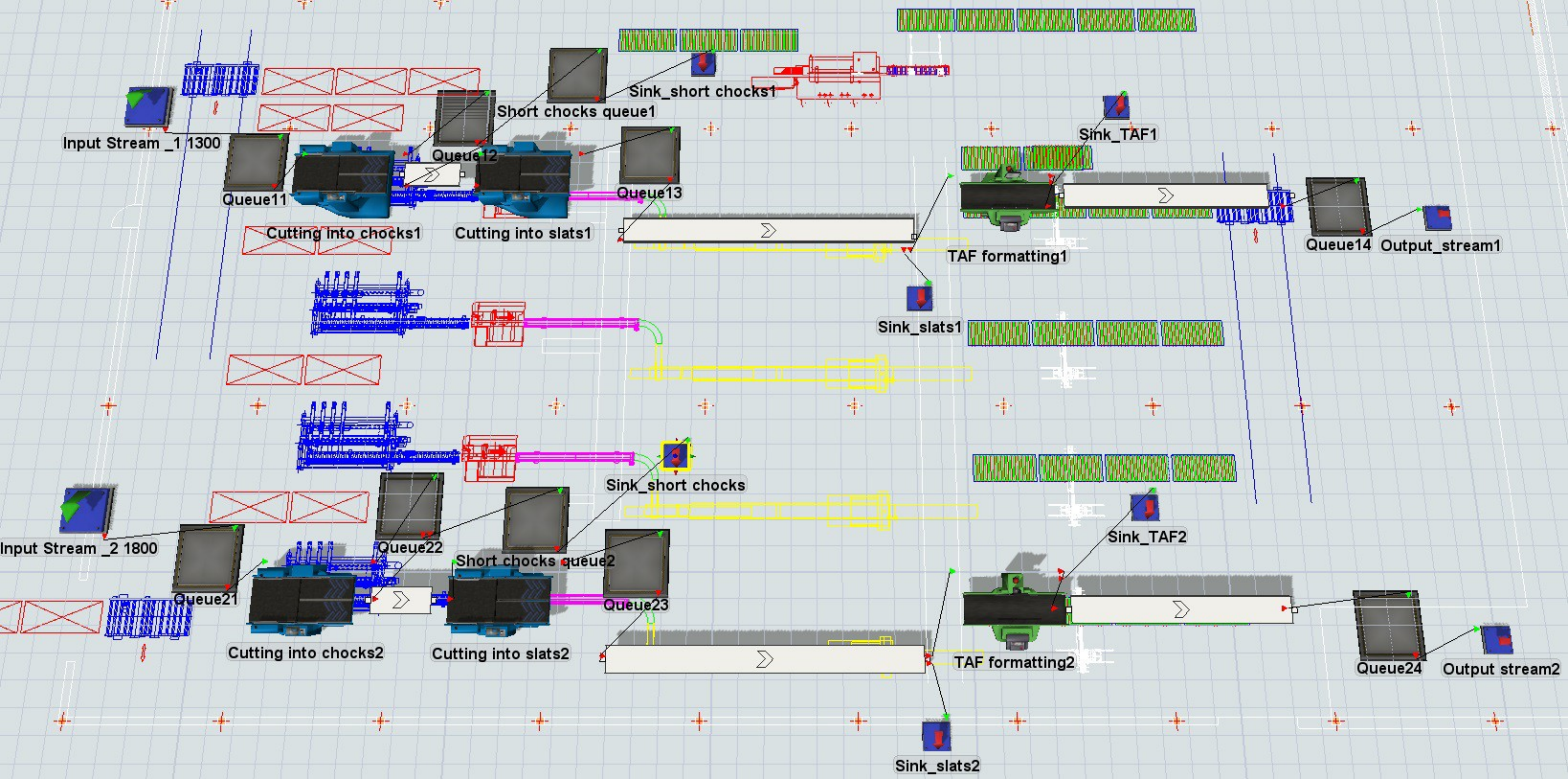
Overview of the simulation model of two production lines created in FlexSim.

The process was modelled in FlexSim, a Discrete Event Simulation environment. An overview of the devised model representation in FlexSim is presented in Fig 4. A production line was created with workstations and their technological parameters as in the actual processes. The production line could process two types of input material: 1300 mm and 1800 mm long timber. The simulation ended when all material was fully processed. Processing time and geometric parameters of the machines were taken from technical documentation and verified with the maintenance department.

Machines were connected with conveyors of constant velocity. All aforementioned data was implemented in a FlexSim model using dedicated objects. In some cases, object logic was adjusted using flex script code that allowed flexible modification of object behaviour in the model.

Volume of the input and output was calculated by multiplying all three linear dimensions (items were considered cuboids) and measured at every processing stage.

Three groups of experiments were conducted, which varied in number of raw timbers: 50, 100 and 200 pieces. Due to the stochastic approach, 30 independent replications of each scenario were executed.

### Description of statistical fitting

The performance of the manufacturing process depends on many input variables, some of which might be considered as random and be described by either empirical or known statistical distributions. In the modelling of geometric inaccuracies, we assumed the following properties of input material to be stochastic:

- length,
- width,
- thickness.

In the process, the initial raw material in a form of roughly cut timbers is transformed into formatted elements of more regular geometry. In this study, it was considered two processes as statistically independent because the dimensions of the output material changes significantly. This can be supported by the fact that no correlations were found for most common mathematical relations (linear, exponential and polynomial), which is quantified as R^2^< 0.05 for all the measured data samples.

The loss of material due to insufficient quality of produced slats from which the final floor module is formed, was also taken into consideration. This was modelled as binomial distribution, with probability of success (a slat meets the desired quality) equal to 0.95.

**Fig 5:**
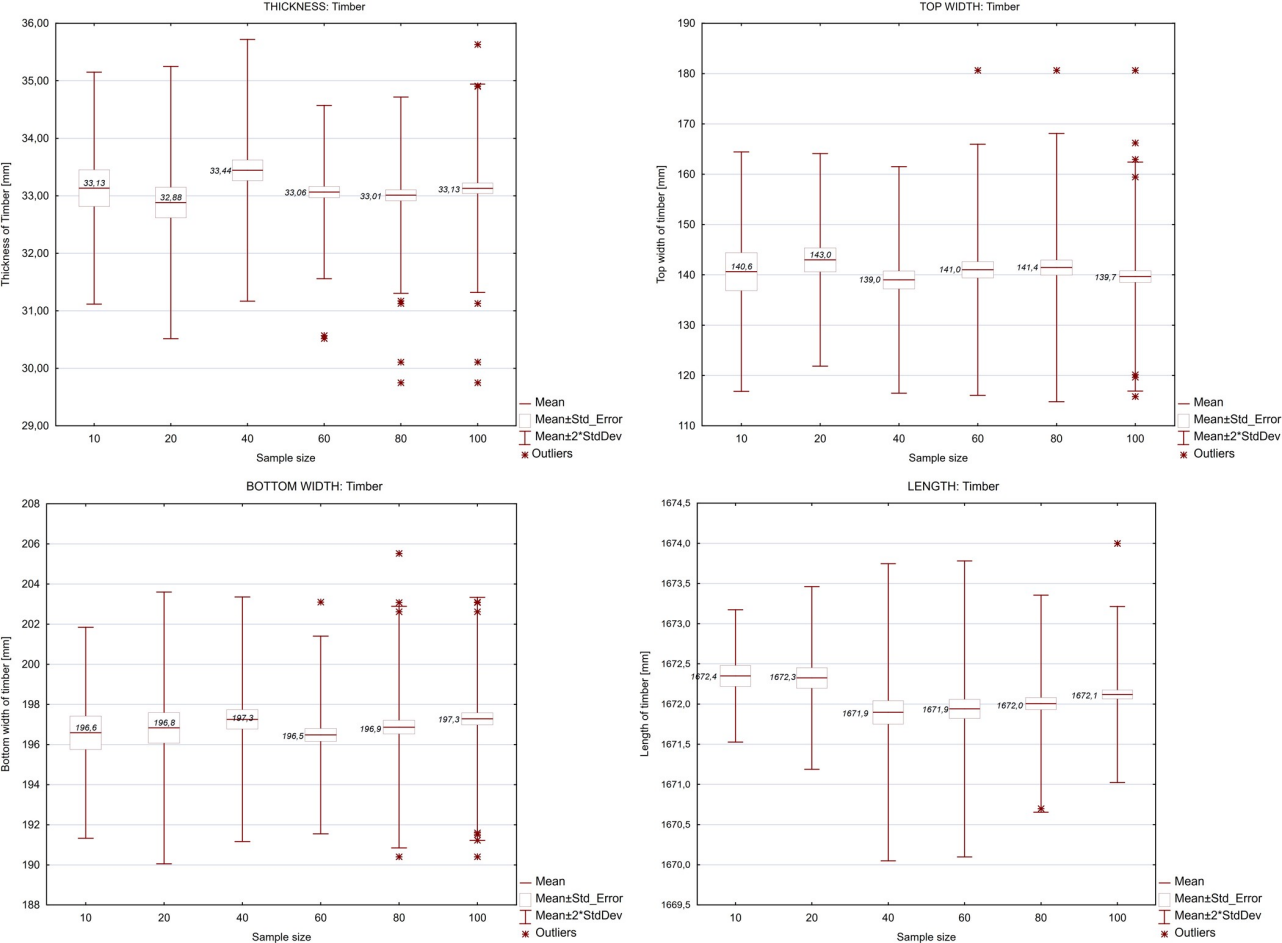
Characteristics of the geometric dimensions of the input material for different sample sizes, taking into account in the simulation model.

The input data was the geometric dimensions of the product and semi-finished products, which was collected manually using validated measuring instruments. Fig 5 shows the statistics of these characteristics (thickness, width, length) of the semi-finished and final product.

The input data was then used to build statistical distributions for geometric dimensions of input material in ExpertFit software [16]. This determined the best-fit model based on the empirical data using well-known statistical tests (Anderson–Darling, Kolmogorov, Chi-Square) for 29 various continuous or discrete distributions. In cases where goodness of fitness is insufficient, application of empirical distribution are suggested. From a simulation perspective, it is important to note that, the software provides the exact representation of the statistical distribution (fitted or empirical) that can be easily implemented in discrete event simulation environment. We took only the best-fitted distribution of the empirical data into consideration. If none of the available distributions could be fitt (assumed p < 0.05 for all of the nonparametric tests), an empirical distribution was used instead. Results of distribution fitting are shown in Table 1. Notation of distributions using location, scale and shape parameters is presented in Table 2.

**Table 1:** Fitted distributions to empirical data for formatted input material.

Number of samples used for distribution fitting	Nominal length of input material	Length	Width	Thickness
**100**	1300	empirical	loglaplace(147.627, 7.723, 12.093)	loglaplace(0.000, 30.180, 342.320)
	1800	empirical	loglaplace(148.392, 6.988, 9.680)	loglogistic(0.000, 30.165, 469.893)
**80**	1300	lognormal2(1229.205, 33.173, 0.041)	loglaplace(153.513, 1.887, 3.320)	loglaplace(0.000, 30.160, 285.626)
	1800	empirical	loglaplace(152.158, 3.247, 5.746)	loglaplace(0.000, 30.170, 303.571)
**60**	1300	weibull(0.000, 1262.954, 877.125)	loglaplace(153.593, 1.817, 3.378)	loglogistic(0.000, 30.121, 355.425)
	1800	weibull(1670.085, 2.109, 2.000)	loglaplace(153.577, 1.773, 3.378)	loglaplace(0.000, 30.120, 272.805)
**40**	1300	loglogistic(0.000, 1262.497, 1285.724)	loglogistic(148.392, 7.188, 13.380)	loglaplace(0.000, 30.120, 206.915)
	1800	empirical	loglaplace(153.149, 2.291, 5.251)	loglaplace(0.000, 30.120, 206.915)
**20**	1300	beta(1253.491, 1266.606, 12.0515, 5.546)	johnsonbounded(151.686, 155.786, -2.323, 1.318)	beta(29.309, 30.496, 11.598, 6.448)
	1800	invertedweibull(0.000, 1672.208, 2498.743)	johnsonbounded(154.664, 157.670, 0.574, 0.847)	loglogistic(0.000, 30.074, 435.126)
**10**	1300	loglogistic(1259.013, 3.497, 2.831)	johnsonbounded(153.000, 158.940, 0.194, 0.895)	johnsonbounded(28.056, 30.351, -2.382, 1.004)
	1800	pearsont6(1668.800, 7.909, 42.574, 99.998)	weibull(0.000, 156.693, 268.110)	loglaplace(0.000000, 30.040, 299.616)

**Table 2:** Description of the distributions used in the study (in Table 1).

Used name in Table 1	Distribution	Parameters [23–25]
loglaplace	Log-Laplace	*γ*, *β*, *α*
loglogistic	Log-logistic	*γ*, *β*, *α*
lognormal2	Lognormal	*γ*, *β*, *α*
pearsont6	Pearson type VI	*γ*, *β*, *α1*, *α2*
weibull	Weibull	*γ*, *β*, *α*
invertedweibull	Inverted Weibull	*γ*, *β*, *α*
beta	Beta	*a*, *b*, *α1*, *α2*
johnsonbounded	Johnson Single Bounded	*a*, *b*, *α1*, *α2*

## Results

The aim of the analysis was to determine the effectiveness of forecasting or calculating loss of wooden material in the technological process by means of simulation experiments. The study also concerned the influence of the sample size of input data constituting the basis for generating input data for modelling. The reference was the actual data obtained in the technological process.

The research shows that the variability of loss for both types of material (1300 mm and 1800 mm), calculated by the simulation based on the data starting from the 40-element sample, is low.

**Fig 6:**
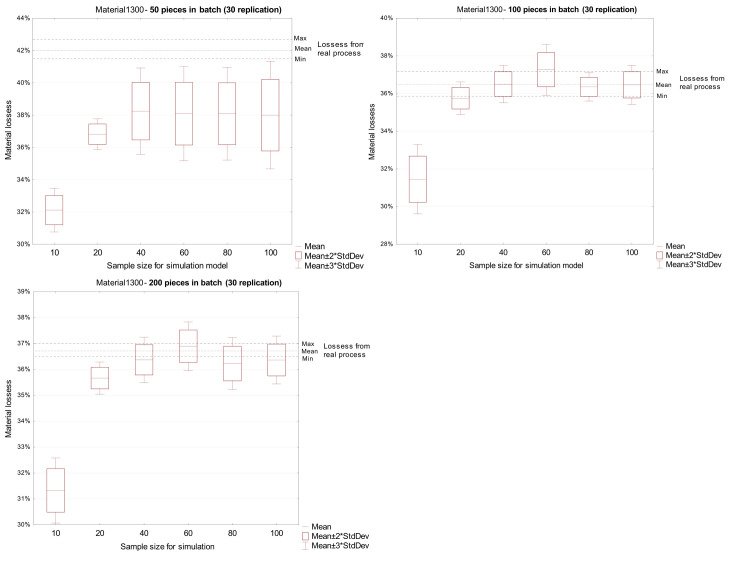
Comparison of loss from a real process with loss received in the simulation model for material 1300 mm.

The results obtained for 1300 mm material were underestimated in relation to the actual results for each tested production batch size and each variant of the input dataset. Fig 6 shows the comparative results for three production batches, where solid lines are the actual data, and the point marks are from the simulation.

**Fig 7:**
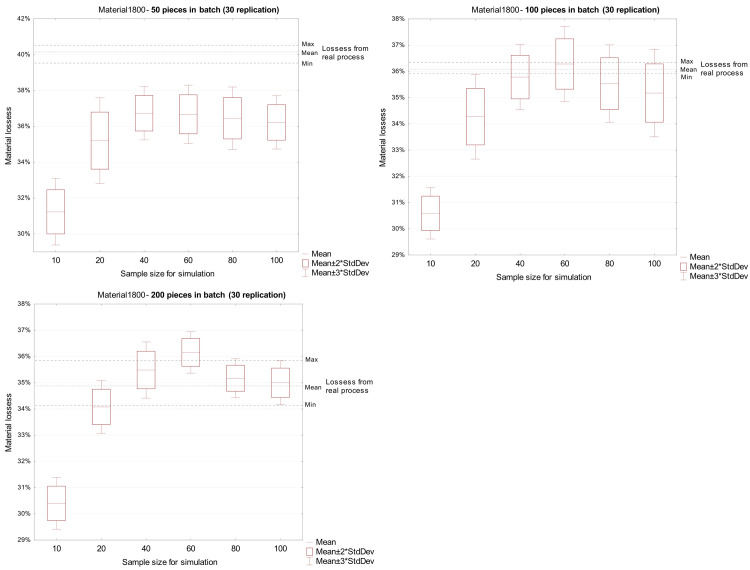
Comparison of loss from a real process with loss received in the simulation model for material 1800 mm.

Regarding 1800 mm material, the material loss from the simulation experiments were overestimated when compared with actual values for a production batch of 100 and 200 pieces (Fig 7).

Simulation experiments allow determination the influence of the size of the statistical sample used in the simulation model on the forecasting of material loss in the discussed process. There was no significant effect on the accuracy of the results obtained in the simulation model for samples of 40 elements or more. These results apply to all types of material (1300 and 1800 mm timbers) and all analyzed production batch sizes (50, 100 and 200 pieces).

The misestimation of material loss by simulation is caused by the dispersion of the geometric parameters of the input material. In simulation experiments where the value of the standard deviation was greater than in the case of the batch of material subject to calculation, the value of material loss was greater than the actual loss. Conversely, a lower value of the standard deviation of geometric quantities in the experiments underestimated the size of loss (Table 3).

**Table 3:** The values of the standard deviation of the geometric dimensions of the input and validation material.

Geometric characteristic	Input data for model						Validating data		
	Sample size used for distribution fitting						Production batch quantity		
	10	20	40	60	80	100	50	100	200
Material 1300									
Thickness	1,01	1,18	1,14	0,75	0,85	0,91	0,64	0,66	0,63
Top width	11,90	10,56	11,26	12,48	13,33	11,37	11,19	12,60	12,05
Bottom width	2,63	3,39	3,05	2,46	3,01	3,03	3,49	3,14	3,15
Length	0,4	0,6	0,9	0,9	0,7	0,5	0,4	0,6	0,8
Material 1800									
Thickness	1,71	1,83	1,33	1,25	1,29	1,37	1,39	1,40	1,37
Top width	12,54	11,56	11,70	11,55	10,60	10,82	11,29	11,20	11,45
Bottom width	2,83	3,39	2,77	2,99	2,55	2,61	2,81	2,82	2,94
Length	8,24	7,89	7,87	7,82	7,28	7,54	7,28	7,95	7,89

The quality of the simulation model is significantly affected by the quality and size of the collected sample, based on which the stochastic model is estimated. It is generally assumed that the sample should consist of independent observations and should be large enough to sufficiently reveal the nature and variation of the analyzed population. In industrial practice, this is often difficult because the data collection process may be long or the variability of the analyzed feature is so large that the quality of developed simulation models may be low. Such a situation takes place in the discussed wood processing process, in which the geometric heterogeneity of the input material is high. The presented results indicate that the decision regarding the size of the sample is crucial as it can ensure the expected quality of the model.

The analysis of the presented results leads to the conclusion that the insufficient sample size in the case of data from the wood processing process does not allow capture of the process variability. On the other hand, a large sample size, i.e. 80 or more observations of naturally heterogenous material, collected in this process takes into account outliers, which may lower the prognostic quality of the model.

At all stages, the acquisition and analysis of input data is an important part of the simulation process. An incorrect decision related to the sample size on the basis of which the statistical model is identified, may lead to a low-quality model, and result in miscalculation of forecasted material loss. Based on the conducted simulation experiments, the recommended sample size, for which a reliable model for simulating material loss in the production of the middle layer of a wooden floorboard can be developed, ranges from 40 to 60 independent measurements.

Creating the credible model for forecasting of the material loss (without interfering with the manufacturing technology) is a way to increase the efficiency of production through better material planning in the whole manufacturing process. Knowing that the improvement in material efficiency (understood as the yield from the input material) by 1% per annum can provide, in the presented case, savings of about 250,000 EUR, the simulation model of the material loss can be of significant importance. Most importantly, minimizing the loss of natural material such as wood also contributes to reduction of the negative impact of this sector on the environment—minimizing the felling of trees, waste and greenhouse gas emissions resulting from waste disposal.

## Conclusion

Determining the loss of input materials is a very important issue in the implementation of many production processes. This knowledge allows determination of the demand for raw materials, production planning and calculation of manufacturing cost. This task is particularly difficult in the case of raw materials that are dimensionally and structurally heterogeneous, such as wood. This article presents research on the use of simulation to determine material loss in the production of floor board components. The analysis of the results shows that simulation experiments can form the basis for the calculation of material loss. However, this requires the preparation of appropriate data as the basis for the calculation. As the results show, the size of the sample constituting the basis for the simulation experiments does not significantly affect the final result. It is possible to determine the cardinality that allows the stability state of the simulation model to be obtained. However, it is important, to adopt the appropriate characteristics of the sample representing a larger population—ensuring the representativeness of measurable parameters. Inappropriate selection causes underestimation or overestimation of the results.

Based on the available literature, there exist no guidelines or industrial standards related to minimal sample size for analysis, modelling or simulation of material loss in wood processing. This study addresses that problem and helps to better understand the consequences of geometric inhomogeneity of the wood for manufacturing.

Forecasting of material loss in processes, the main cause of which is the variability of the input material, is difficult to assess in industrial practice. Therefore, creating a simulation model is rationally justified. The presented model was implemented in a large manufacturing company and describes the actual material loss more reliably than the former assumption that the input material is homogeneous.

The obtained research results indicate the potential for further research, especially in the field of greater efficiency of mapping the dimensional characteristics of the samples constituting the basis for the implementation of simulation experiments and creating simulation models taking into account criteria such as: wood class and origin, distribution of gnarls or knots, and much more, which will significantly increase the prognostic quality of the model.
